# 4-Hydroxy Hexenal Derived from Docosahexaenoic Acid Protects Endothelial Cells via Nrf2 Activation

**DOI:** 10.1371/journal.pone.0069415

**Published:** 2013-07-23

**Authors:** Atsushi Ishikado, Katsutaro Morino, Yoshihiko Nishio, Fumiyuki Nakagawa, Atsushi Mukose, Yoko Sono, Nagisa Yoshioka, Keiko Kondo, Osamu Sekine, Takeshi Yoshizaki, Satoshi Ugi, Takashi Uzu, Hiromichi Kawai, Taketoshi Makino, Tomio Okamura, Masayuki Yamamoto, Atsunori Kashiwagi, Hiroshi Maegawa

**Affiliations:** 1 Department of Medicine, Shiga University of Medical Science, Shiga, Japan; 2 Department of Diabetes, Metabolism and Endocrinology, Kagoshima University Graduate School of Medical and Dental Sciences, Kagoshima, Japan; 3 Osaka Laboratory, JCL Bioassay Corporation, Osaka, Japan; 4 Research & Development Department, Sunstar Inc., Osaka, Japan; 5 Department of Pharmacology, Shiga University of Medical Science, Shiga, Japan; 6 Department of Medical Biochemistry, Tohoku University Graduate School of Medicine, Miyagi, Japan; Max Delbrueck Center for Molecular Medicine, Germany

## Abstract

Recent studies have proposed that n-3 polyunsaturated fatty acids (n-3 PUFAs) have direct antioxidant and anti-inflammatory effects in vascular tissue, explaining their cardioprotective effects. However, the molecular mechanisms are not yet fully understood. We tested whether n-3 PUFAs showed antioxidant activity through the activation of nuclear factor erythroid 2-related factor 2 (Nrf2), a master transcriptional factor for antioxidant genes. C57BL/6 or Nrf2^−/−^ mice were fed a fish-oil diet for 3 weeks. Fish-oil diet significantly increased the expression of heme oxygenase-1 (HO-1), and endothelium-dependent vasodilation in the aorta of C57BL/6 mice, but not in the Nrf2^−/−^ mice. Furthermore, we observed that 4-hydroxy hexenal (4-HHE), an end-product of n-3 PUFA peroxidation, was significantly increased in the aorta of C57BL/6 mice, accompanied by intra-aortic predominant increase in docosahexaenoic acid (DHA) rather than that in eicosapentaenoic acid (EPA). Human umbilical vein endothelial cells were incubated with DHA or EPA. We found that DHA, but not EPA, markedly increased intracellular 4-HHE, and nuclear expression and DNA binding of Nrf2. Both DHA and 4-HHE also increased the expressions of Nrf2 target genes including HO-1, and the siRNA of Nrf2 abolished these effects. Furthermore, DHA prevented oxidant-induced cellular damage or reactive oxygen species production, and these effects were disappeared by an HO-1 inhibitor or the siRNA of Nrf2. Thus, we found protective effects of DHA through Nrf2 activation in vascular tissue, accompanied by intra-vascular increases in 4-HHE, which may explain the mechanism of the cardioprotective effects of DHA.

## Introduction

N-3 polyunsaturated fatty acids (n-3 PUFAs) such as eicosapentaenoic (EPA) and docosahexaenoic acids (DHA) in fish oil were shown to reduce cardiovascular disease in epidemiological studies of Eskimo during the 1970s [1], [2]. More recent large-scale interventions and cross-sectional studies have shown that n-3 PUFAs reduce cardiovascular events independently of the classical risk factors for atherosclerosis [3]–[6], which suggests direct anti-atherogenic effects of n-3 PUFAs on vascular tissues. Many studies have already demonstrated that n-3 PUFAs display a variety of bioactive actions such as anti-inflammatory [7], [8] and antioxidant effects [9], [10], improvement of endothelial function [11], [12] and a suppressive effect on monocyte adhesion in vascular tissue [8], [13], [14] explaining the anti-atherogenic effects of n-3 PUFA. In a recent study, enzymatic metabolites from n-3 PUFAs, including resolvins and protectins, were reported to exert potent anti-inflammatory effects in aortic endothelial cells, leading to atheroprotective effects [15]. It has also been suggested that activation of peroxisome proliferator-activated receptors (PPARs) by n-3 PUFAs in vascular endothelial cells plays an important role in the atheroprotective effects of n-3 PUFAs [16], [17]. A more recent study has shown that DHA suppresses NFκB activation through G protein-coupled receptor 120 (GPR120) in macrophages [18]. However, the critical mechanism explaining the cardioprotective effects of n-3 PUFAs remains a matter of debate.

Nuclear factor erythroid 2-related factor 2 (Nrf2) is a redox-sensitive master regulatory transcriptional factor, and plays an important role in maintaining the atheroprotective capacity of vascular endothelial cells by regulating endothelial redox balance [19]–[21]. In unstimulated cells, Nrf2 resides in the cytoplasm bound to Kelch-like ECH-associated protein 1 (Keap1). Electrophiles, shear stress or reactive oxygen species (ROS) instigate modification of the cysteine residues of Keap1, which allows translocation to the nucleus and binding to the antioxidant response element (ARE) consensus sequence, resulting in the transcription of antioxidant enzymes such as heme oxygenase-1 (HO-1), γ-glutamyl-cysteine ligase (GCL), and NAD(P)H quinone oxidoreductase 1 (NQO1) [22]–[25]. HO-1 in particular, a rate-limiting enzyme in heme metabolism, has been recognized as an important factor protecting vascular tissue against atherosclerosis by exerting antioxidant, anti-inflammatory, anti-proliferative, anti-apoptotic and vasodilatory effects on the vasculature [26].

A recent study reported that a fish-oil diet induced HO-1 expression in the kidney of obstructive renal injury rats [27]. DHA has also been shown to increase HO-1 expression in a Nrf2-dependent manner in EA.hy926 cells [28]. Furthermore, it has been reported that the suppressive effects of n-3 PUFAs such as EPA and DHA on the LPS-induced inflammatory response were absent in peritoneal macrophages isolated from Nrf2 null mice [29], and further studies have proposed that J3 and D4 isoprostanes (as oxidation products of n-3 PUFAs) activate Nrf2 in HepG2 and porcine pulmonary endothelial cells, respectively [30], [31]. However, it is uncertain whether intake of a fish-oil diet activates Nrf2 in vascular tissue, resulting in the vasculoprotective effect.

In the present study, we tested the hypothesis that fish-oil diet increases antioxidant enzymes such as HO-1, an important atheroprotective factor, and the endothelium-dependent vasodilatory response, through the activation of Nrf2 in vascular tissue. We also investigated whether fish-oil diet increases the intra-vascular concentrations of the peroxidation end-product of n-3 PUFAs to discuss the biological mechanisms on the vasculoprotective effects. Furthermore, we investigated the mechanisms of EPA and DHA on Nrf2-mediated induction of antioxidant enzymes including HO-1 and the consequent antioxidant effect in human umbilical vein endothelial cells (HUVECs).

## Materials and Methods

### Reagents

Acetylcholine (ACh) was from Daiichi-Sankyo (Tokyo, Japan). Sodium nitroprusside (SNP) and fatty acid-free BSA were purchased from Nacalai Tesque (Kyoto, Japan). Papaverine hydrochloride was from Nichi-iko (Toyama, Japan). 2′,7′-Dichlorofluorescin diacetate (H2DCFDA) and peroxidase-linked anti-mouse antibody were purchased from Invitrogen (Grand Island, NY). EPA, DHA, 4-hydroxy hexenal (4-HHE), NS-398 and SC-58125 were purchased from Cayman (Ann Arbor, MI). L-phenylephrine, butylated hydroxytoluene (BHT), *tert*-butyl hydroperoxide (tBHP), 3-(4,5-dimethyl-2-thiazolyl)-2,5-diphenyl-2H-tetrazolium bromide (MTT), zinc protoporphyrin (ZnPP), indomethacin, α-tocopherol and N-acetylcysteine (NAC) were obtained from SIGMA-ALDRICH (St. Louis, MO). Anti-HO-1 antibody was from Assay design (Ann Arbor, MI). Anti-β-actin antibody was purchased from Cell Signaling (Danvers, MA). Anti-Nrf2 antibody (H-300), anti-lamin A/C antibody and peroxidase-linked anti-rabbit antibody were purchased from Santa Cruz Biotechnology (Santa Cruz, CA). Anti-GCLM antibody was purchased from Abcam (Cambridge, UK). Anti-p62 antibody was obtained from BD Biosciences (Franklin Lakes, NJ). Anti-GAPDH antibody (MAB374) was from Millipore (Billerica, MA).

### Animals

All animal experiments were approved by the Shiga University of Medical Science Committee on Animal Research (Permit Number: 2009-11-3). Male C57BL/6 mice were purchased from CLEA Japan, Inc. C57BL/6 background Nrf2*^−/−^* mice have been previously described [32]. A Fish oil (#112246) or soy oil-based control diet (#110700, AIN-93 G, Dyets Inc., Bethlehem, PA) was fed to each mouse. Table 1 shows the detailed composition of each experimental diet. Table 2 also shows fatty acid composition of dietary fats used for the experimental diets. Mice were euthanized by isoflurane overdose after 3 weeks of diet, following an overnight fast, to analyze the effect of each diet on mRNA or protein expression of HO-1 in thoracic aorta.

**Table 1:** Composition of the experimental diets.

Constituents (g/kg)	Control diet	Fish-oil diet
Casein	200.0	200.0
Corn starch	397.5	227.4
Sucrose	100	–
Maltose dextrin	132.0	132.0
Cellulose	50.0	50.0
t-Butylhydroquinone	0.014	0.068
Vitamin mix	10.0	10.0
Mineral mix	35.0	35.0
L-Cystine	3.0	3.0
Choline bitartrate	2.5	2.5
Soybean oil	70.0	70.0
Menhaden oil	–	270.0

**Table 2:** Fatty acid composition (wt%) of dietary fats.

Fatty acid	Soybean oil	Menhaden oil
14∶0	–	9.0
15∶0	–	0.7
16∶0	10.2	17.1
16∶1	–	12.5
16∶2	–	1.7
16∶3	–	1.7
16∶4	–	1.8
17∶0	–	0.9
18∶0	4.5	2.8
18∶1	22.7	11.4
18∶2 n-6	54.8	1.5
18∶3 n-3	7.8	1.6
18∶4 n-3	–	3.5
20∶0	–	0.2
20∶1	–	1.6
20∶4 n-3	–	1.4
20∶4 n-6	–	0.9
20∶5 n-3	–	15.5
21∶5 n-3	–	0.8
22∶1	–	0.5
22∶5 n-3	–	2.4
22∶6 n-3	–	9.1
24∶1	–	0.1
unknown	–	1.3

### Endothelium-dependent Vasorelaxation

C57BL/6 and Nrf2*^−/−^* mice were euthanized by 160 mg/kg pentobarbital sodium salt after 3 weeks of diet, following an overnight fast, to analyze the effect of each diet on the endothelium-dependent vasodilatory response in the thoracic aorta. The thoracic aorta was isolated and cut into rings with special care to reserve the endothelium. The rings, under a resting tension of 1.0 g, were exposed to 30 mM KCl; this contraction was defined as 100% for subsequent analysis of contraction in response to agonists. Responses to cumulative concentrations of L-phenylephrine (10^−7^ to 10^−6^ M) were then obtained. Relaxant responses to ACh (10^−9^ to 10^−7^ M) or SNP (10^−9^ to 10^−6^ M) were obtained in the rings pre-contracted with L-phenylephrine to construct concentration-response curves. At the end of each experiment, 10^−4^ M papaverine was added to induce the maximal relaxation, which was taken as 100% for comparison with relaxation induced by agonists.

### Assay for Measuring Intra-aortic 4-HHE and 4-hydroxy Nonenal (4-HNE)

Intra-aortic 4-HHE and 4-HNE were quantitatively analyzed using a liquid chromatography-tandem mass spectrometry (LC-MS/MS). Thoracic aortas were fractured in liquid nitrogen using a Cryo Press (Microtec Co., Ltd., Chiba, Japan) and subjected to lipid extraction. The samples solubilized in 490 µL chloroform/methanol (1∶1, v/v) and 10 µL BHT solution (10 mg/mL in ethanol) were incubated at 36°C for 1 h. Total 4-HHE and 4-HNE levels were quantitatively measured as previously reported [33]. Briefly, [^2^H_3_]-4-HNE (Cayman) as internal solution was spiked to 200 µL samples obtained from lipid extraction. Then, samples were passed through using a mix-mode anion exchange solid-phase extraction (SPE) cartridge, Oasis MAX (Waters, Milford, MA). After extraction, the eluate was spiked with BHT solution and acidified cyclohexanedione (CHD) reagent was added to derivatize 4-HHE or 4-HNE. CHD-derivatization was carried out at 60°C for 1 h, and derivatized 4-HHE or 4-HNE was then extracted using a SPE cartridge, Oasis HLB (Waters). The eluate from SPE was injected into a LC-MS/MS system. LC was performed using an ACQUITY UPLC (Waters), and an API4000 triple quadrupole tandem mass spectrometer (AB Sciex, Foster City, CA) was used as a detector. Chromatographic separation was achieved using a reversed-phase column, ACQUITY CSH C18 (Waters), with two mobile phases and electrospray ionization in positive ionization with selected reaction monitoring (SRM) mode was used to analyze derivatized 4-HHE and 4-HNE. 4-HHE or 4-HNE levels were normalized to the weight of tissue.

### Assay for Measuring Intra-aortic DHA and EPA

Intra-aortic DHA and EPA were also quantitatively analyzed using a LC-MS/MS. Total DHA and EPA levels were quantitatively measured as previously reported [34]. Briefly, internal solution containing [^2^H_5_]-DHA (Cayman) and [^2^H_5_]-EPA (Cayman) was spiked to all samples (20 µl) obtained from lipid extraction as described above and calibration standard samples, and the samples were evaporated by nitrogen gas. Following the addition of acetonitrile/6N HCl (90/10, v/v), samples were incubated at 100°C for 45 min. Finally, liquid-liquid extraction with ethyl acetate was performed and the reconstituted samples were injected into an optimized LC-MS/MS system. LC was performed using an ACQUITY UPLC [a YMC-Triart C18 (YMC Co., Ltd., Kyoto, Japan), and a liner gradient combination of two mobile phases] and an API4000 triple quadrupole tandem mass spectrometer was used as a detector using atmospheric pressure chemical ionization in negative ionization with SRM mode. DHA and EPA levels were normalized to the weight of tissue.

### Cell Culture

HUVECs were cultured according to a previously reported method [35], [36]. When cells reached confluence, they were starved overnight in medium containing 2% FBS before their use for the experiments on 4-HHE analysis, real-time RT-PCR, western blotting and Nrf2 activity. Fatty acids or other reagents were dissolved in medium supplemented with 2% FBS for the treatment.

### Fatty Acid Treatment

DHA or EPA was administered as a complex with fatty acid-free BSA. DHA or EPA (0.30 mM each) was dissolved in ethanol (2.5 mL), and gradually solubilized in 8.4% BSA solution (14.3 mL) at 37°C. BSA-conjugated fatty acids were dissolved in serum-containing medium at the final desired concentration. The control medium containing BSA was prepared in a similar manner. 4-HHE was dissolved in ethanol, and further dissolved in serum-containing medium. Ethanol was used as a control for 4-HHE.

### Assay for Measuring Intracellular 4-HHE and 4-HNE

Intracellular 4-HHE and 4-HNE were extracted in 13.2 mM BHT-containing RIPA buffer (Thermo Scientific, Rockford, IL), and subjected to LC-MS/MS analysis as described above. 4-HHE or 4-HNE levels were normalized to the protein concentration in the lysate.

### RNA Extraction and Real-time PCR Analysis

Total RNA was extracted from cells using a Total RNA Mini Kit (Bio-Rad, Hercules, CA). Thoracic aortas were fractured in liquid nitrogen using a Cryo Press and subjected to RNA extraction. Single-stranded cDNA was synthesized from 0.5 µg of total RNA using a PrimeScript RT Reagent Kit (Takara Bio, Shiga, Japan), and endogenous genomic DNA was degraded by DNase I (Invitrogen). Quantitative analyses of HO-1, GCL, modifier subunit (GCLM), sequestosome 1 (SQSTM1, p62) and Nrf2 mRNAs were performed by real-time PCR using the ABI 7500 Fast Real-Time PCR System (Applied Biosystems, Japan). Premix Ex Taq (Takara Bio) and Assay-on-Demand, Gene Expression Products [mice; Mm00516007_m1 for HO-1 and Mm00607939_s1 for β-actin; HUVECs; Hs01110250_m1 for HO-1, Hs00157694_m1 for GCLM, Hs00177654_m1 for p62, Hs00232352_m1 for Nrf2 and Hs02387368_g1 for ribosomal protein S18 (RPS18) (Applied Biosystems, Foster City, CA)] were used for quantitative real-time PCR analysis. Quantitative data *in vivo* or *in vitro* were respectively normalized to the expression level of β-actin and RPS18.

### Western Blotting Analysis

Thoracic aortas were fractured in liquid nitrogen using a Cryo Press and then solubilized in RIPA buffer (Thermo Scientific) containing protease inhibitors (Thermo Scientific). Whole cell lysate from HUVECs was prepared in RIPA buffer containing protease inhibitors. Nuclear lysates were prepared using a Nuclear/Cytosol Fractionation Kit (BioVision, Mountain View, CA) according to the manufacturer’s protocol. The lysates were denatured by boiling in SDS sample buffer (Thermo Scientific), resolved by SDS-PAGE and then transferred to a nitrocellulose membrane by electroblotting. Blots were then incubated with a rabbit anti-HO-1 primary antibody, a rabbit anti-β-actin primary antibody, a rabbit anti-Nrf2 primary antibody, a rabbit anti-lamin A/C primary antibody, a mouse anti-GCLM primary antibody, a mouse anti-p62 primary antibody, or a mouse anti-GAPDH primary antibody plus a horseradish peroxidase-linked secondary antibody, and detected by chemiluminescence using an ImageQuant LAS 4000mini system (GE Healthcare, Japan).

### Cell Transfection and Luciferase Assay

The ARE-luciferase reporter plasmid (pGL4.27(Nrf2-luc2P/minP/Hygro)) was obtained from Promega (Madison, WI). HUVECs (3.4×10^4^) were plated in 12-well type I collagen-coated plates for 48 h. Cells were cotransfected with 0.2 µg luciferase expression plasmid and 0.05 µg of pRL-TK plasmid (Promega) as a normalization reference for transfection efficiency using SuperFect Transfection Reagent (Qiagen, Valencia, CA) for 8 h, then cells were stimulated with DHA, EPA or 4-HHE for 16 h. Cells were harvested, and Firefly and *Renilla* luciferase activities were determined using a Dual-Luciferase Reporter Assay System (Promega) with a luminometer (GloMax 20/20n, Promega).

### Nrf2 DNA Binding Assay

Nrf2 activation was assayed using Active Motif’s (Carlsbad, CA) enzyme-linked immunosorbent assay (ELISA)-based transactivation TransAM kit, following the manufacturer’s protocol. Nrf2 from nuclear lysate, which specifically binds to its consensus oligonucleotide, was analyzed colorimetrically using a spectrophotometer at 450 nm.

### Transfection with Small Interfering (si)RNA

HUVECs were plated in type I collagen-coated plates until 80–90% confluency. siRNA against Nrf2 or PPARα was used to silence Nrf2 or PPARα, respectively (On-TARGET plus SMARTpool Reagent; Thermo Scientific). A control siRNA was also used (On-TARGET plus Non-targeting siRNA #1, Thermo Scientific). HUVECs were transfected with 20 nM of Nrf2 siRNA, PPARα siRNA or control siRNA using DharmaFECT 1 siRNA Transfection Reagent (Thermo Scientific) and incubated for 24 h in medium containing 2% FBS, after which the medium was refreshed. After incubation for a further 24 h, HUVECs were stimulated with DHA for 6 h to analyze the effects on mRNA expression. To analyze protein expression, 32 h after transfection, cells were stimulated with DHA or 4-HHE for 16 h, and then subjected to western blotting analyses. The silencing effects of Nrf2 and PPARα were confirmed by real-time RT-PCR or western blotting analyses.

### MTT Measurement of Cell Viability

HUVECs were seeded on 24-well type I collagen-coated plates. To determine the preventive effect of DHA on tBHP-induced cell toxicity, confluent cells were pretreated with DHA (37.5–75 µM) for 16 h, washed with phosphate-buffered saline (PBS), and exposed to tBHP (250 or 500 µM) for 6 h. Cell viability was determined by conventional MTT reduction assay. MTT is a tetrazolium salt cleaved to formazan by the mitochondrial respiratory chain enzyme, succinate dehydrogenase. After treatment with tBHP, cells were incubated with MTT solution (0.5 mg/mL) in culture medium for 3 h. The culture medium was then removed, the formazan product was solubilized in dimethylsulfoxide, and the absorbance at 577 nm was measured using a microplate reader. Values were expressed as percentage of cell survival. Absorbance from tBHP-untreated cells was set at 100%. To determine the involvement of Nrf2 in oxidative stress-induced cell viability, cells were pretreated for 16 h with DHA (75 µM) or 4-HHE (5–10 µM) 32 h after transfection with Nrf2 siRNA, and then exposed to tBHP for 6 h.

### Intracellular GSH and GSSG Assay

Intracellular GSH and GSSG levels were measured colorimetrically using a glutathione assay kit (GSH/GSSG-412, OxisResearch, Foster City, CA) according to the manufacturer’s protocol, and normalized to protein concentration in the lysate.

### Assay for Measuring ROS

Intracellular ROS production was determined using a fluorescence probe, H2DCFDA. Confluent HUVECs in 24-well culture plates were incubated with 75 µM DHA for 16 h before treatment with 20 µM H2DCFDA for 20 min. Following a PBS wash, cells were either untreated, or treated with 250 or 500 µM tBHP in medium containing 2% FBS. The fluorescence released from cells was recorded immediately at 492 nm (excitation) and 525 nm (emission) using a fluorescent microplate reader at different time intervals over a 2-h period.

### Lactate Dehydrogenase (LDH) Cytotoxicity Assay

LDH activity was measured using an LDH Cytotoxicity Assay Kit (Cayman), according to the manufacturer’s protocol.

### Statistical Analysis

Data are presented as mean ± SE. Differences between more than three groups were analyzed by two-tailed multiple *t*-test with Bonferroni correction. Comparisons between two groups were analyzed using a two-tailed Student’s *t*-test. Statistical significance was established at *P*<0.05.

## Results

### Nrf2-mediated Effects of Fish-oil Diet in Aorta

**Figure 1:**
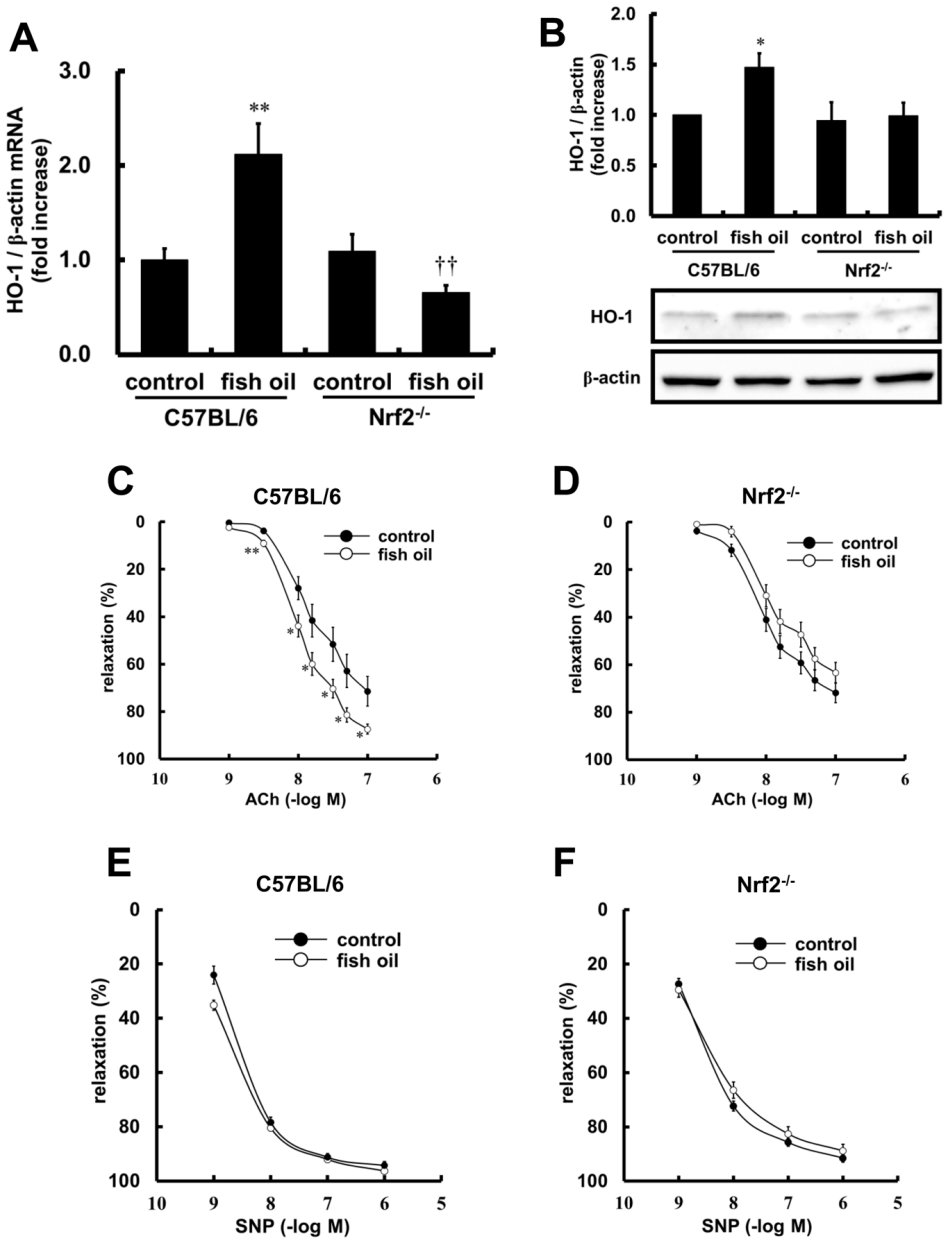
Effects of fish-oil diet on HO-1 expression and endothelium-dependent vasorelaxation in thoracic aortas. Control or fish-oil diet was fed to C57BL/6 or Nrf2*^−/−^* mice for 3 weeks. (A) The relative mRNA expressions of HO-1 in thoracic aortas were analyzed quantitatively using real-time RT-PCR. Each value represents the mean ± SE of 6–10 animals. (B) Total cell lysates from thoracic aortas were subjected to western blotting analyses. Each value represents the mean ± SE of four animals. (C–F) Concentration-vasodilatory response curves induced by ACh (C, D) or SNP (E, F) in aortic rings obtained from C57BL/6 (C, E) or Nrf2*^−/−^* mice (D, F) fed with fish-oil diet for 3 weeks. Each value represents the mean ± SE of 12–18 rings. *P<0.05, **P<0.01, compared with control diet-fed C57BL/6 mice. **^††^** *P*<0.01, compared with fish-oil diet-fed C57BL/6 mice.

To investigate the Nrf2-mediated effects of a fish-oil diet in vascular tissue, we examined expression of the HO-1 gene. The fish-oil diet-fed groups showed a smaller increase in body weight compared with the control diet-fed group, but there was no difference between C57BL/6 and Nrf2*^−/−^* mice (Table 3). Plasma triglyceride was significantly increased in both the Nrf2*^−/−^* groups, but there were no significant differences in plasma glucose, total cholesterol or non-esterified fatty acid (NEFA) among the four groups (Table 4). Fig. 1A shows the effects of a fish-oil diet on HO-1 mRNA expression in thoracic aorta tissue of C57BL/6 or Nrf2*^−/−^* mice. The fish-oil diet significantly increased mRNA expression of HO-1 in C57BL/6 mice, while the increase was not observed in Nrf2*^−/−^* mice. The increase in HO-1 protein expression by fish-oil diet was similarly absent in the Nrf2*^−/−^* mice as shown in Fig. 1B. Next, we examined whether the fish-oil diet altered ACh-dependent vasorelaxation in the aorta. The fish-oil diet significantly increased the ACh-dependent vasodilatory response compared with control diet in wild type mice. In contrast, there was no effect of the fish-oil diet in Nrf2*^−/−^* mice (Fig. 1C and 1D). The effects of fish-oil diet on SNP-dependent vasorelaxation were also investigated. Fish-oil diet had no effect on SNP-dependent response (Fig. 1E and 1F). Thus, the data indicate that a fish-oil diet increases the Nrf2-mediated vasodilatory response in an endothelium-dependent manner.

**Table 3:** Body weight changes of C57BL/6 (n = 10) and Nrf2^−/−^ mice (n = 6) fed with fish-oil diet for 3 weeks.

	C57BL/6 (g)		Nrf2^−/−^ (g)	
	Controldiet	Fish-oildiet	Controldiet	Fish-oildiet
Baseline	24.2±0.7	24.7±0.6	24.1±1.0	25.2±0.5
After 3 weeks	27.9±0.9	26.1±0.7	27.5±2.0	26.2±0.4

Each value represents the mean ± SE.

**Table 4:** Plasma characteristics of C57BL/6 (n = 10) and Nrf2^−/−^ mice (n = 6) fed with fish-oil diet for 3 weeks.

	C57BL/6		Nrf2^−/−^	
	Controldiet	Fish-oildiet	Controldiet	Fish-oildiet
Blood glucose (mM)	2.86±0.33	2.87±0.28	2.50±0.32	2.85±0.11
Triglyceride (mM)	0.82±0.10	0.65±0.11	1.35±0.08**	1.44±0.13**
Total cholesterol (mM)	2.58±0.28	2.23±0.15	2.73±0.41	2.84±0.20
NEFA (µEq/L)	812.9±130.5	688.2±80.9	836.8±62.5	803.3±95.2

Each value represents the mean ± SE.

** *P*<0.01, compared with the corresponding group of C57BL/6 mice.

### Effects of Fish-oil Diet on Concentrations of DHA, EPA and the Peroxidation Products in Aortic Tissue

**Figure 2:**
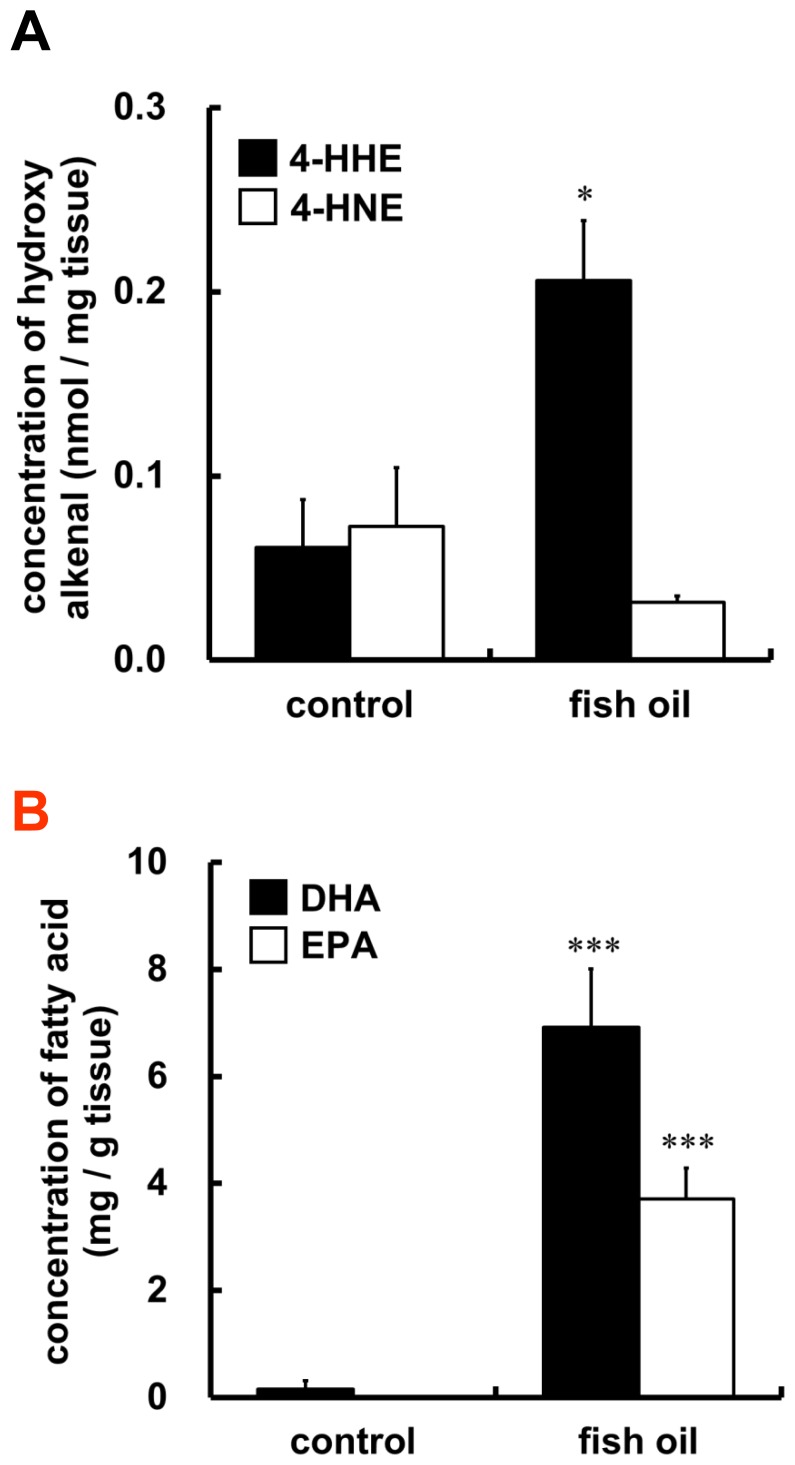
Effects of fish-oil diet on concentrations of 4-HHE, 4-HNE, DHA and EPA in thoracic aortas. Control or fish-oil diet was fed to C57BL/6 mice for 3 weeks. The concentrations of intra-aortic 4-HHE or 4-HNE (A), and DHA or EPA (B) were measured by LC-MS/MS analyses. Each value represents the mean ± SE of 4 animals. *P<0.05, ***P<0.001, compared with each corresponding control.

Intra-aortic concentrations of 4-HHE, an end-product of n-3 PUFAs peroxidation, as well as 4-HNE, an end-product of n-6 PUFAs peroxidation, were measured after dietary fish-oil supplementation for 3 weeks, using LC-MS/MS analyses. Fish-oil diet significantly increased intra-aortic concentration of 4-HHE in C57BL/6 mice, whereas that of 4-HNE tended to be decreased (Fig. 2A). We also measured intra-aortic concentrations of DHA or EPA by LC-MS/MS analyses. Fish-oil diet increased intra-aortic concentration of DHA rather than EPA (Fig. 2B), despite lower concentration of DHA than EPA in menhaden oil used for the experimental diet (Table 2).

### Effects of DHA and EPA on Intracellular 4-HHE or 4-HNE Production in Vascular Endothelial Cells

**Figure 3:**
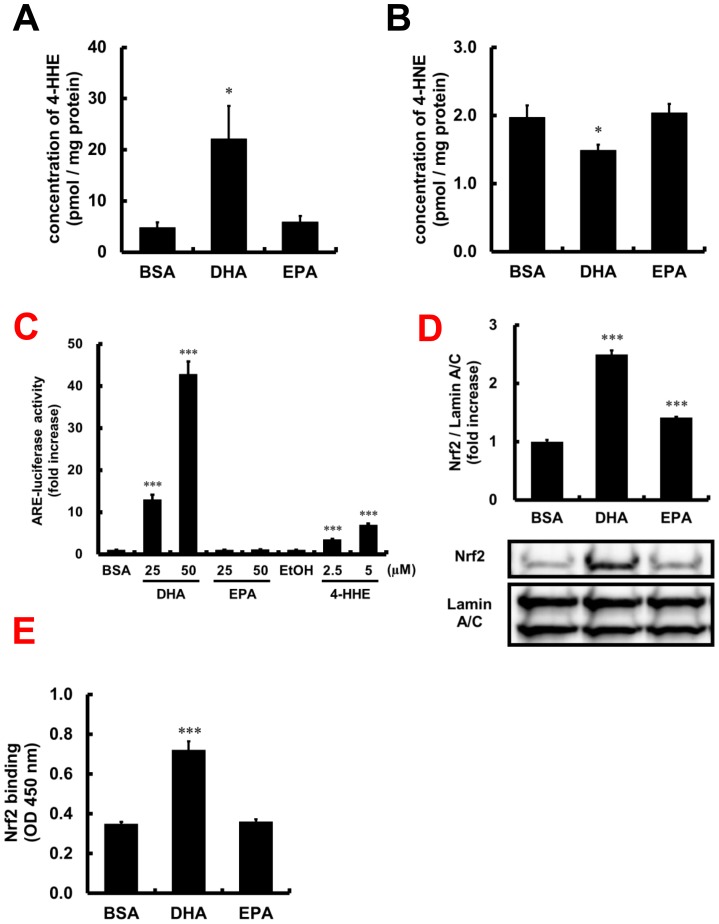
Effect of DHA on intracellular 4-HHE and 4-HNE levels, or Nrf2 activation in HUVECs. (A, B) HUVECs were incubated with 75 µM DHA or EPA for 6 h. The concentrations of intracellular 4-HHE or 4-HNE were measured by LC-MS/MS analyses. Each value represents the mean ± SE of 6–8 experiments. (C) HUVECs were cotransfected with a reporter plasmid (pGL4.27(Nrf2-luc2P/minP/Hygro)) and a control plasmid (pRL-TK). After transfection, HUVECs were incubated with DHA, EPA or 4-HHE for 16 h. The ratio (reporter/control luciferase activity) obtained from control cell lysate was set at 1. Each value represents the mean ± SE of four experiments. (D, E) HUVECs were incubated with 75 µM of DHA or EPA for 6 h. (D) Nuclear lysates were subjected to western blotting analyses. Each value represents the mean ± SE of three experiments. (E) Analysis of the binding of Nrf2 in nuclear lysates to its consensus oligonucleotide was performed using the ELISA-based TransAM Nrf2 kit. Each value represents the mean ± SE of four experiments. *P<0.05, ***P<0.001, compared with each corresponding control.

Intracellular concentrations of 4-HHE and 4-HNE were measured 6 h after the treatment of HUVECs with DHA or EPA, using LC-MS/MS analyses. Fig. 3A and 3B show their respective intracellular concentrations. Cells incubated with DHA showed a significant increase in intracellular 4-HHE, and a significant decrease in 4-HNE. In contrast, EPA did not affect the intracellular levels of 4-HHE or 4-HNE. The stock solution of BSA-conjugated DHA used for the experiment was confirmed to be free of 4-HHE, suggesting that the production of these peroxidation products occurred in the cells (data not shown).

### Activation of Nrf2 by DHA or EPA

**Figure 4:**
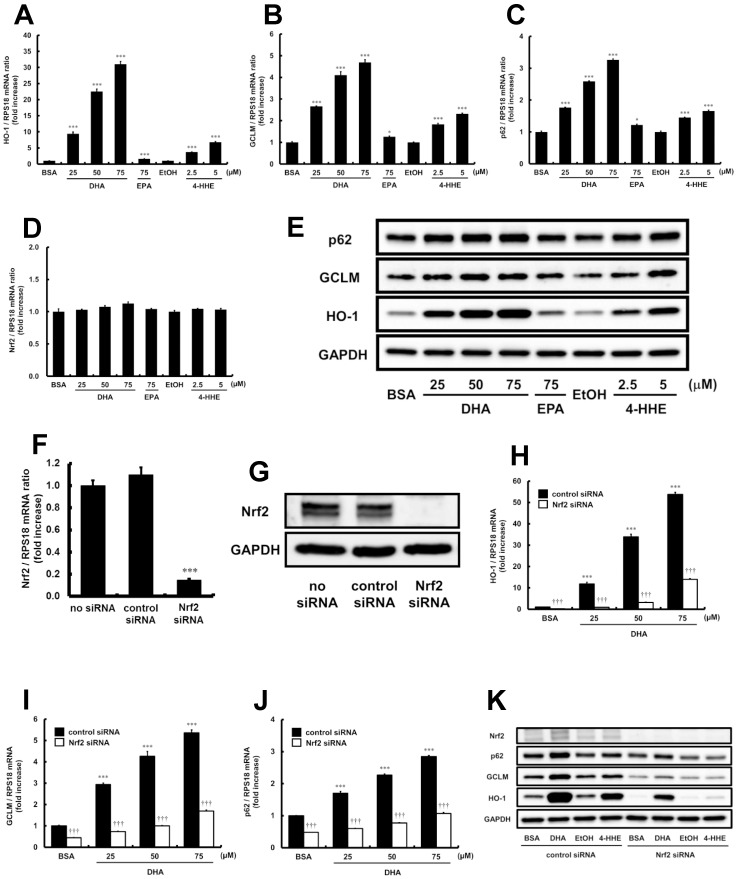
Effects of DHA, EPA or 4-HHE on HO-1, GCLM or p62 expression in HUVECs. (A–D) HUVECs were incubated with DHA, EPA or 4-HHE for 6 h. The relative mRNA expressions of HO-1, GCLM, p62 or Nrf2 were quantitated using real-time RT-PCR. Each value represents the mean ± SE of 3 experiments. (E) HUVECs were incubated with DHA, EPA or 4-HHE for 6 h. Total cell lysates were subjected to western blotting analyses. (F, G) HUVECs were treated with Nrf2 siRNA or control siRNA, and incubated for 48 h. (F) The relative mRNA expression of Nrf2 was analyzed quantitatively using real-time RT-PCR. Each value represents the mean ± SE of four experiments. (G) Whole cell lysates were subjected to western blotting analyses. (H–K) HUVECs were transfected with Nrf2 siRNA or control siRNA. (H–J) After 48 h, the cells were incubated with DHA for a further 6 h. The relative mRNA expressions of HO-1, GCLM or p62 were quantitated using real-time RT-PCR. Each value represents the mean ± SE of 3 experiments. (K) 32-h after transfection, the cells were incubated with DHA (50 µM) or 4-HHE (5 µM) for a further 16 h. Whole cell lysates were subjected to western blotting analyses. *P<0.05, ***P<0.001, compared with each corresponding control, ^†††^ *P*<0.001, compared with the corresponding cells treated with control siRNA.

Luciferase activities of DHA, EPA and 4-HHE were evaluated in HUVECs using an ARE-loaded luciferase expression plasmid. DHA showed significant and dose-dependent ARE-luciferase activity, as did 4-HHE. However, EPA had no effect on the luciferase activity (Fig. 3C). Translocation of Nrf2 to the nucleus was evaluated by western blotting analysis of Nrf2 in the nuclear fraction. As shown in Fig. 3D, treatment with DHA increased Nrf2 in the nuclear lysate, while the effect of EPA was much lower than that of DHA. The binding activity of Nrf2 to its consensus oligonucleotide was significantly increased by stimulation with DHA, but not increased by stimulation with EPA (Fig. 3E). However, DHA and 4-HHE had no effect on Nrf2 mRNA expression (Fig. 4D).

### Effects of DHA and EPA on Expressions of Nrf2 Target Gene

Because DHA but not EPA clearly activated Nrf2, the effects of DHA, EPA and 4-HHE on the expression of Nrf2 target genes were investigated in HUVECs. As shown in Fig. 4A, 4B and 4C, both DHA (25–75 µM) and 4-HHE (2.5–5 µM) dose-dependently increased the mRNA expressions of HO-1, GCLM and p62, those of which contain an ARE in their genes. DHA and 4-HHE also markedly increased the protein expressions of HO-1, GCLM and p62 (Fig. 4E). The impact of EPA on these effects was much lower than that of DHA at the same concentration.

### Nrf2-dependence on DHA-induced HO-1, GCLM or p62 Expression in HUVECs

To determine the role of Nrf2 in the induction of HO-1, GCLM and p62 by DHA, HUVECs were treated with either Nrf2 siRNA or control siRNA. The expression of Nrf2 mRNA in cells treated with Nrf2 siRNA was reduced by approximately 87% (Fig. 4F), while Nrf2 protein expression in whole cell lysate was also markedly suppressed (Fig. 4G). As shown in Fig. 4H, 4I and 4J, the increases in HO-1, GCLM or p62 mRNA expression caused by DHA were significantly suppressed by Nrf2 knockdown. Similarly, knockdown of Nrf2 also reduced DHA- as well as 4-HHE-induced protein expressions of HO-1, GCLM and p62 (Fig. 4K).

### Preventive Effects of DHA on Oxidative Stress-induced Cellular Damage

**Figure 5:**
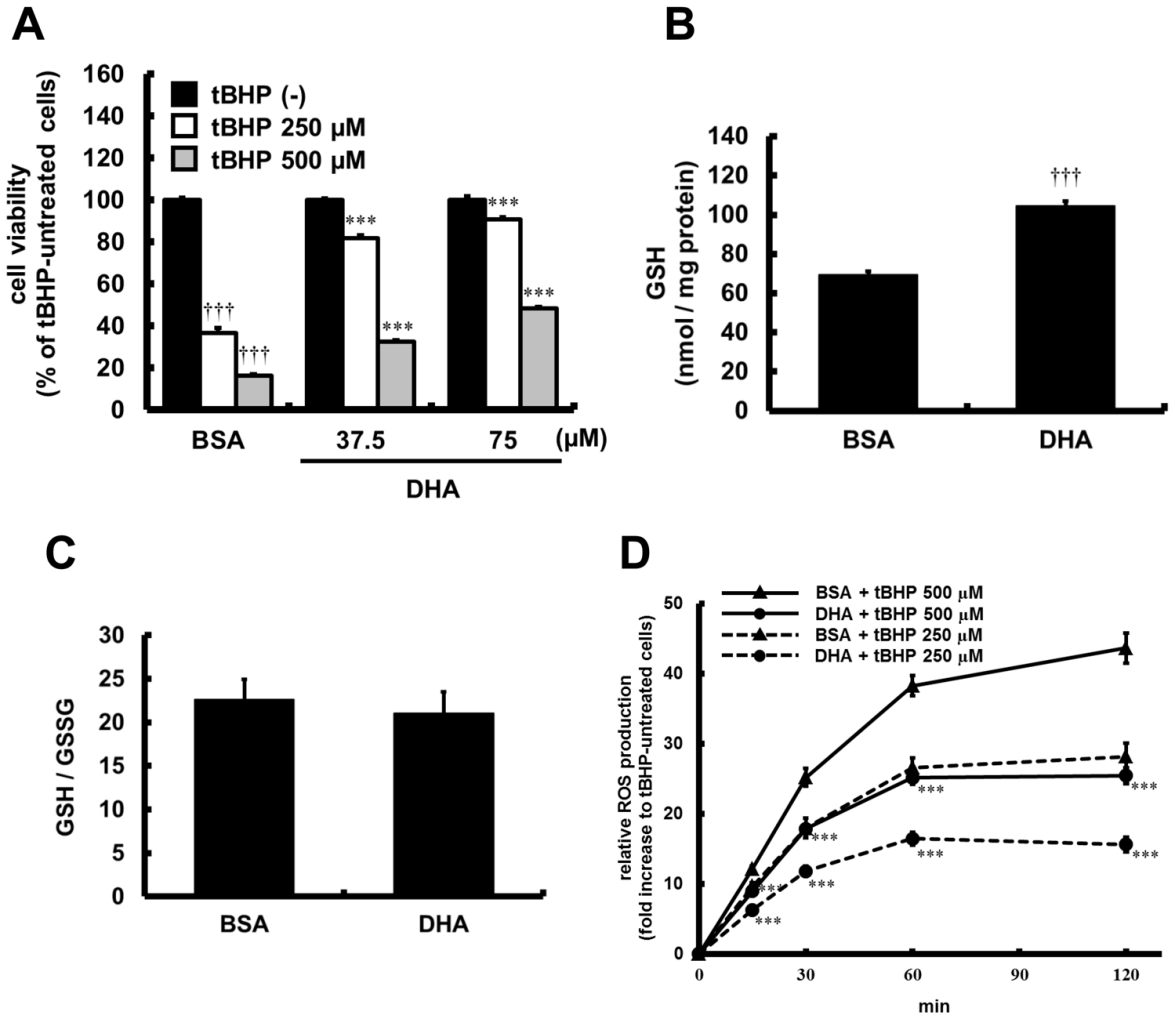
Antioxidant effects of DHA in HUVECs. (A) HUVECs were pretreated with DHA for 16 h, and then stimulated with tBHP (250 or 500 µM) for 6 h. Cell viability was determined by MTT assay. Values are expressed as percentage of cell survival, and each represents the mean ± SE of 4 experiments. (B, C) HUVECs were incubated with DHA (75 µM) for 16 h. GSH concentration (B) and ratio of GSH/GSSG (C) were determined. Each value represents the mean ± SE of 7 experiments. (D) HUVECs were pretreated with DHA (75 µM) for 16 h, and exposed to tBHP (250 or 500 µM). ROS released from cells was determined at different time intervals over 2-h period. Each value represents the mean ± SE of 8 experiments. ***P<0.001, compared with tBHP-treated BSA control, **^†††^** *P*<0.001, compared with tBHP-untreated BSA control.

Cytoprotective effects of DHA against tBHP-induced oxidative stress were investigated in HUVECs. Fig. 5A shows cell viability assessed using the MTT assay 6 h after the induction of oxidative stress by tBHP. Treatment with tBHP (250 and 500 µM) showed dose-dependent cell toxicity, while pretreatment with DHA for 16 h significantly protected against tBHP-induced cytotoxicity dose-dependently.

### Effects of DHA on Redox Homeostasis or Oxidative Stress-induced ROS Production in HUVECs

Fig. 5B and 5C show the intracellular GSH concentration and the GSH/GSSG ratio, respectively. Treatment with DHA for 16 h significantly increased intracellular GSH concentration 1.5 fold compared with BSA control without changing the GSH/GSSG ratio. Furthermore, to investigate the ability of DHA to reduce oxidative stress, we assessed ROS production from tBHP-treated HUVECs. Treatment with tBHP (250 and 500 µM) increased ROS production dose-dependently as shown in Fig. 5D, while pretreatment with DHA for 16 h significantly suppressed the intracellular ROS production caused by both concentrations of tBHP tested.

### HO-1- or Nrf2-dependence on Antioxidant Effects of DHA in HUVECs

**Figure 6:**
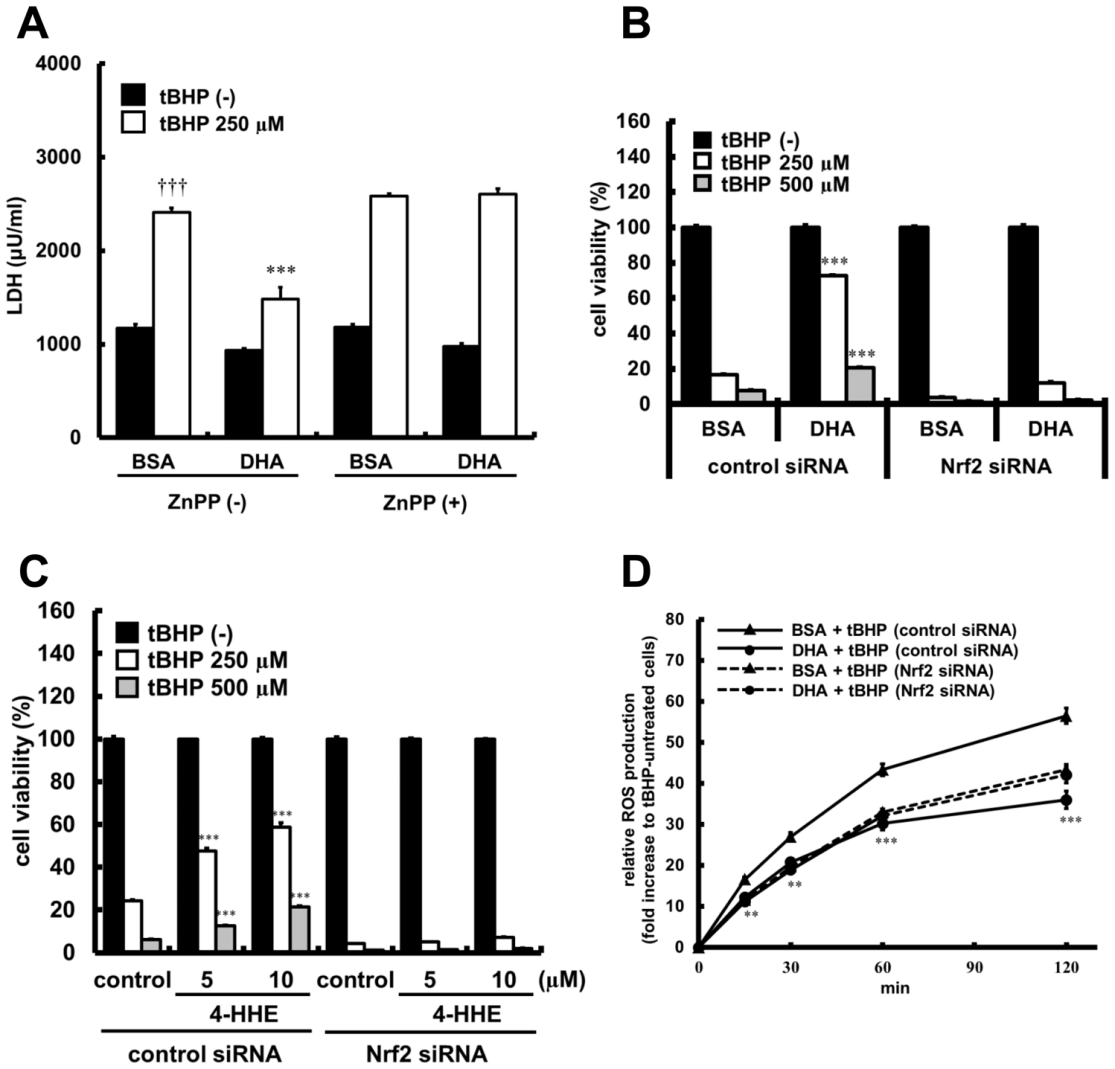
Effects of HO-1 inhibition and Nrf2 knockdown on antioxidant effects of DHA or 4-HHE. (A) HUVECs were pretreated in the presence of DHA (75 µM) with or without ZnPP (5 µM) for 16 h, and stimulated with tBHP (250 µM) for 24 h. LDH in the supernatant of culture media was quantitatively analyzed. Each value represents the mean ± SE of 6 experiments. (B–D) HUVECs were transfected with Nrf2 siRNA and control siRNA, respectively. 32-h later, they were incubated with DHA (75 µM) or 4-HHE (5 or 10 µM) for additional 16 h, and then exposed to tBHP (250 or 500 µM). (B, C) Cell viability 6-h after tBHP treatment was measured by MTT assay. Values are expressed as percentage of cell survival, and each represents the mean ± SE of 3–4 experiments. (D) tBHP (500 µM)-induced ROS release was measured at different time intervals over 2-h period. Each value represents the mean ± SE of 4 experiments. **P<0.01, ***P<0.001, compared with tBHP-treated corresponding control, **^†††^** *P*<0.001, compared with tBHP-untreated BSA control.

To determine the role of HO-1 activity in the antioxidant effects of DHA, HUVECs were treated with ZnPP, an inhibitor of HO-1 activity. Treatment with 250 µM tBHP for 24 h increased LDH leakage into the culture medium, and DHA showed preventive effects on tBHP-induced LDH leakage. Under these conditions, the effect of DHA was abolished by treatment with 5 µM ZnPP (Fig. 6A). In addition, HUVECs treated with the siRNA targeted against Nrf2 were exposed to tBHP (250 or 500 µM) after treatment with DHA for 16 h. As shown in Fig. 6B and 6D, pretreatment of DHA significantly suppressed tBHP-induced cell toxicity or ROS production in the cells treated with control siRNA, but these effects of DHA were disappeared in the cells treated with the siRNA for Nrf2. Furthermore, 4-HHE showed similar Nrf2-mediated effects of DHA (Fig. 6C), suggesting that 4-HHE derived from DHA protected cells from oxidative stress through Nrf2-HO-1 signaling.

### Investigation of the Molecular Mechanisms of DHA-induced HO-1 Expression in HUVECs

**Figure 7:**
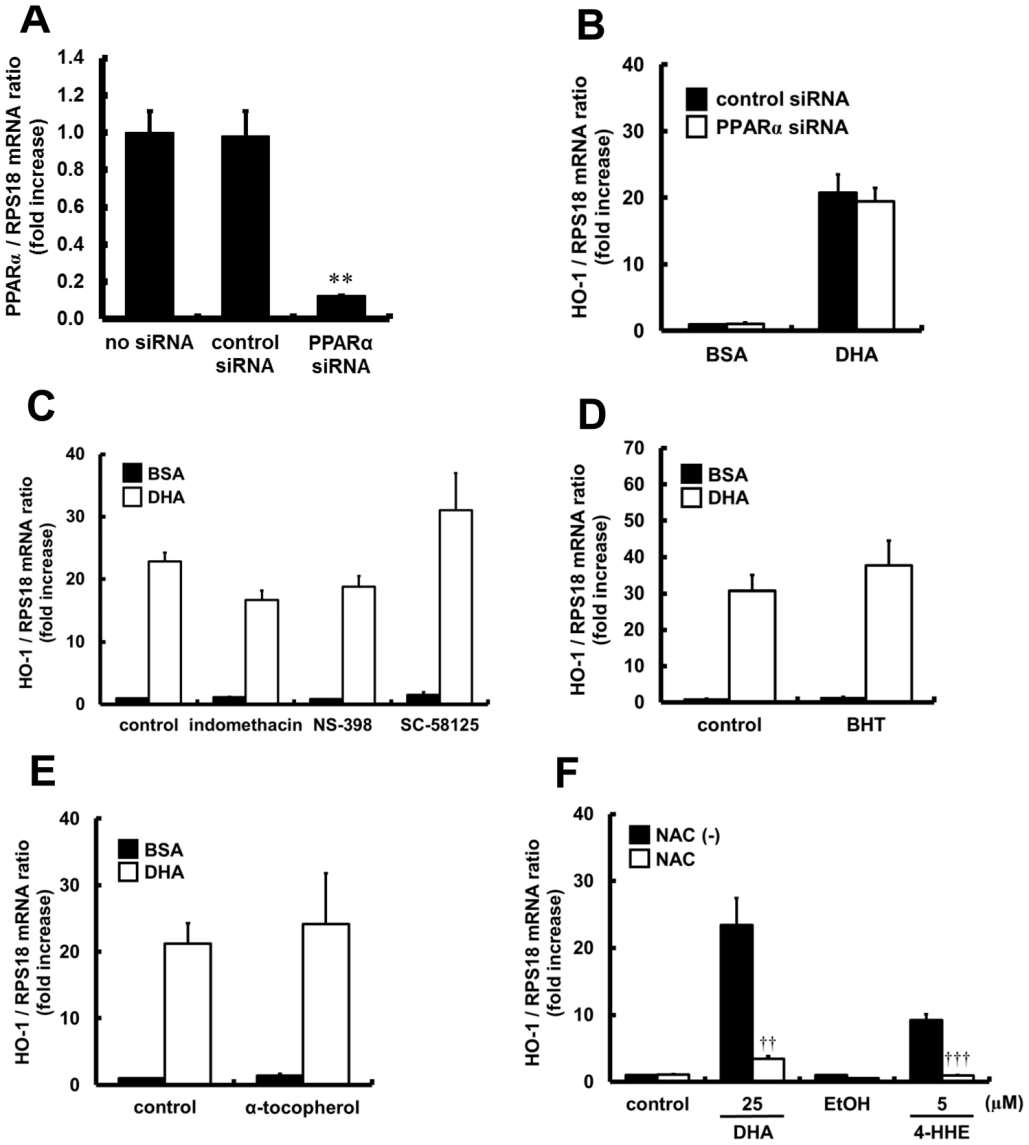
Effects of PPARα siRNA, COX inhibitors or antioxidants on DHA- or 4-HHE-induced HO-1 mRNA expression. (A) HUVECs were treated with PPARα siRNA or control siRNA, and incubated for 48 h. The relative mRNA expression of PPARα was quantitated using real-time RT-PCR. Each value represents the mean ± SE of three experiments. (B) HUVECs were transfected with siRNA targeted against PPARα or control siRNA. After 48 h, the cells were incubated with DHA (25 µM) for a further 6 h. The relative mRNA expression was analyzed quantitatively using real-time RT-PCR. Each value represents the mean ± SE of three experiments. (C–F) HUVECs were pretreated in the presence of indomethacin (10 µM), NS-398 (1 µM), SC-58125 (1 µM), BHT (100 µM), α-tocopherol (100 µM) or NAC (5 mM) for 1 h, and stimulated with DHA (25 µM) or 4-HHE (5 µM) for 6 h. The relative mRNA expression was quantitated using real-time RT-PCR. Each value represents the mean ± SE of 3–5 experiments. **P<0.01, compared with the control cells treated with control siRNA, **^††^** *P*<0.01, **^†††^** *P*<0.001, compared with NAC-untreated corresponding cells.

To explore the molecular mechanisms of DHA on Nrf2 activation, we investigated the pathway upstream of Nrf2 activation. First, HUVECs were transfected with either PPARα siRNA or control siRNA. Fig. 7A shows the knockdown efficiency of mRNA by PPARα siRNA. The expression of PPARα mRNA in the cells treated with PPARα siRNA was reduced by approximately 87%. However, DHA-induced expression of HO-1 mRNA was not reduced in the PPARα knockdown HUVECs (Fig. 7B). Second, HUVECs were treated with several cyclooxygenase (COX) inhibitors or antioxidants, and DHA-induced expression of HO-1 mRNA was examined. As shown in Fig. 7C–E, inhibitors of COX1 or COX2, as well as radical scavengers such as BHT and α-tocopherol, did not reduce DHA-induced expression of HO-1 mRNA. In contrast, NAC, which is the most bioavailable precursor of glutathione, significantly reduced the expression of HO-1 mRNA induced by DHA or 4-HHE (as shown in Fig. 7F).

## Discussion

Recent studies have shown that dietary fish oil can increase Nrf2 target genes including HO-1 in the heart or kidney [27], [37]. It has also been reported that the suppressive effects of EPA and DHA on LPS-induced expression of inflammatory molecules in isolated peritoneal macrophages were not observed in macrophages isolated from Nrf2^−/−^ mice [29]. Furthermore, more recent study has demonstrated that DHA activates Nrf2-HO-1 pathway, resulting in inhibition of NFκB-mediated endothelial inflammation [28]. Although these studies suggest that n-3 PUFAs might have Nrf2-mediated atheroprotective activities, Nrf2-dependent increases in antioxidant activity and vascular endothelial function by n-3 PUFAs have not been reported. In this study, we demonstrated that a fish-oil diet induced the antioxidant adaptive gene HO-1 in aortic tissue, and enhanced vasodilatory responses in an endothelium-dependent manner through the Nrf2 signaling pathway. This study is the first to show the Nrf2-mediated increase in endothelial function by fish oil *in vivo*.

**Figure 8:**
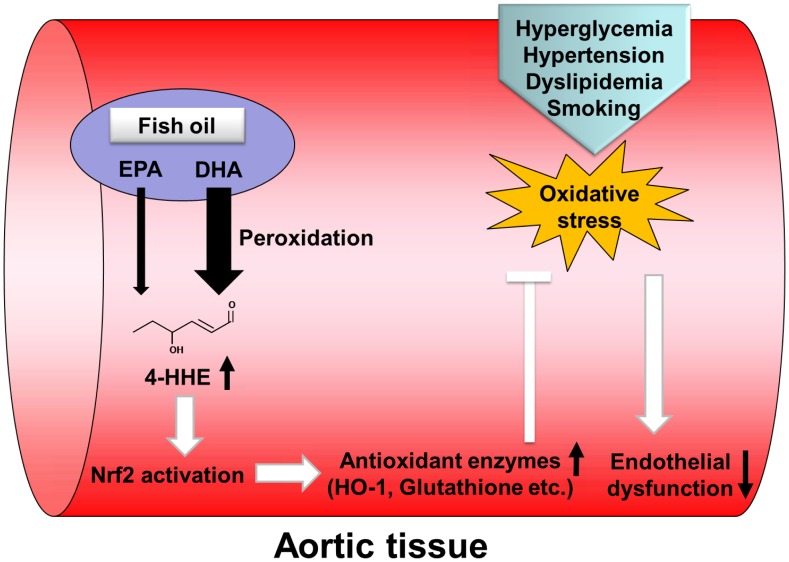
A schematic figure showing the Nrf2-mediated effects of DHA on antioxidant activity and endothelial function in aortic tissue.

We also showed that dietary fish-oil supplementation increases intra-aortic concentration of 4-HHE as an end-product aldehyde of n-3 PUFAs peroxidation, accompanied by intra-aortic predominant increase in DHA rather than that in EPA. In addition, we observed that DHA, but not EPA, causes an increase in intracellular 4-HHE in cultured vascular endothelial cells. Next, we demonstrated that DHA, but not EPA, induces antioxidant enzymes including HO-1 through the activation of Nrf2, resulting in increased antioxidant activity. The present study has also shown that 4-HHE induces HO-1 expression through Nrf2 activation (as reported in our previous study [35]), and increases Nrf2-mediated antioxidant activity as shown in Fig. 6C. It has already been reported that plasma levels of 4-HHE were elevated following supplementation with 800 or 1600 mg/day DHA [38]. Furthermore, protein adducts of 4-HHE have been shown to increase in heart or liver of animals fed an n-3 PUFA-rich diet [37], [39]. Thus, we speculated that 4-HHE derived from DHA might contribute to the vasculoprotective effect of n-3 PUFAs (Fig. 8).

We observed that DHA-induced HO-1 mRNA expression was dramatically reduced by treatment with NAC, probably because cysteine residues in NAC binds to 4-HHE, leading to protection of the cysteine residues in Keap1, a major regulator of Nrf2 protein degradation. We have also shown that BHT or α-tocopherol, as antioxidants, did not affect the DHA-induced mRNA expression of HO-1, suggesting a role of increased intra-vascular 4-HHE by DHA in Nrf2-mediated antioxidant abilities. This is also supported by a recent study showing that 4-HNE, a lipid peroxidation product of n-6 PUFAs, can directly modify cysteine residues on Keap1, leading to activation of Nrf2 [40]. In contrast, recent studies have proposed the participation of the COX- or lipoxygenase-mediated metabolites of n-3 PUFAs, or PPARs and GPR120 in the cardioprotective effects of n-3 PUFAs [15], [16], [18]. Thus, we investigated whether previously reported mechanisms of DHA are responsible for Nrf2-mediated expression of HO-1. However, our results showed that COX or PPARα was of little relevance to DHA-induced HO-1 mRNA expression. We also observed no expression of GPR120 in HUVECs (data not shown). Thus, we speculated that intra-vascular 4-HHE produced from DHA may directly stimulate Keap1-Nrf2 pathway and contribute to the cardioprotective effects of n-3 PUFAs. 4-HHE has been considered to be a toxic lipid peroxidation product similar to 4-HNE, a peroxidation product of n-6 PUFAs [41]. However, a recent study has shown that intravenous administration of 4-HNE protected against cardiac ischemia-reperfusion injury in mice via the Nrf2-dependent pathway [42]. Therefore, it is plausible that lower concentrations of intra-vascular 4-HHE associated with the intake of DHA may have a beneficial effect through the preconditioning effect of Keap1-Nrf2 signaling.

A large scale intervention trial for Japanese with hyper-cholesterolemia has shown that purified EPA can significantly reduce total coronary events in a group with a previous history of such events, despite less of an effect on Nrf2 activation than seen in our study [6]. This implies that mechanisms other than Nrf2 activation contribute to the cardioprotective effects of fish oil-derived n-3 PUFAs. However, recent studies have proposed different beneficial effects between DHA and EPA on cardiovascular risk factors. Sekikawa et al. have reported that, in contrast to EPA, serum DHA levels show a significant inverse correlation to intima-media thickness (IMT) in men aged 40 to 49 years in Japan and the United States [43]. Furthermore, it has been demonstrated that in overweight, mildly hyperlipidemic men, DHA, but not EPA, enhances vasodilatory responses compared with olive oil in the microcirculation of the forearm, although this is predominantly due to endothelial-independent mechanisms [44]. Therefore, it can be considered that Nrf2-mediated antioxidant activity of DHA might contribute to clinical cardioprotective effects in a manner different to that of EPA.

Recent studies have demonstrated that HO-1 plays an important role in preventing atherogenesis *in vivo* [45]–[47]. In the present study, we demonstrated that dietary fish oil induced HO-1 in a manner that was Nrf2-dependent. The antioxidant effects of DHA, as assessed by tBHP-induced LDH leakage in HUVECs, were obviously decreased by treatment of ZnPP, a HO-1 inhibitor, suggesting that Nrf2-HO-1 signaling is a critical factor for the antioxidant effects of DHA. We have already reported that vascular oxidative stress increased by insulin resistance causes decreased tetrahydrobiopterin and increased NADPH oxidase activity, resulting in decreased endothelium-dependent vasorelaxation [48], [49]. In fact, endothelial-specific increases in HO-1 expression have been shown to restore the angiotensin II-induced reduction in ACh-dependent vasorelaxation accompanied by an increase in eNOS phosphorylation [50]. It has also been reported that HO-1 induction by cobalt protoporphyrin, a HO-1 inducer, restores high-blood-flow-dependent remodeling and endothelial function in mesenteric arteries of old rats [51]. Therefore, HO-1-mediated reduction in oxidative stress might contribute to the Nrf2-dependent vasodilatory response induced by fish-oil diet in the present study.

In conclusion, the present study has demonstrated that a fish-oil diet increased HO-1 expression and the endothelium-dependent vasodilatory response through the activation of Nrf2. Furthermore, we have demonstrated that DHA, but not EPA, is able to increase antioxidant activity through Nrf2-mediated HO-1 expression in conjunction with increased intra-vascular 4-HHE. Therefore, the present study provides a possible novel explanation for the cardioprotective effects of DHA.
